# Obesity and incidence of cancer: a large cohort study of over 145 000 adults in Austria

**DOI:** 10.1038/sj.bjc.6602819

**Published:** 2005-10-18

**Authors:** K Rapp, J Schroeder, J Klenk, S Stoehr, H Ulmer, H Concin, G Diem, W Oberaigner, S K Weiland

**Affiliations:** 1 Department of Epidemiology, University of Ulm, Helmholtzstraße 22, 89081 Ulm, Germany; 2 Department of Epidemiology, University of North Carolina, Chapel Hill, NC 27599-7435, USA; 3 Department of Medical Statistics, Informatics and Health Economics, Medical University, Innsbruck, Austria; 4 Agency for Preventive and Social Medicine, Rheinstraße 61, 6900 Bregenz, Austria; 5 Cancer Registry of Tyrol, Department of Clinical Epidemiology of the Tyrolean State Hospitals Ltd, Maximilianstraße 35, 6020 Innsbruck, Austria

**Keywords:** obesity, breast cancer, colonic cancer, endometrial cancer, lymphoma, non-Hodgkin, pancreatic cancer

## Abstract

We investigated the relation of overweight and obesity with cancer in a population-based cohort of more than 145 000 Austrian adults over an average of 9.9 years. Incident cancers (*n*=6241) were identified through the state cancer registry. Using Cox proportional-hazards models adjusted for smoking and occupation, increases in relative body weight in men were associated with colon cancer (hazard rate (HR) ratio 2.48; 95% confidence interval (CI): 1.15, 5.39 for body mass index (BMI) ⩾35 kg m^−2^) and pancreatic cancer (HR 2.34, 95% CI: 1.17, 4.66 for BMI>30 kg m^−2^) compared to participants with normal weight (BMI 18.5–24.9 kg m^−2^). In women, there was a weak positive association between increasing BMI and all cancers combined, and strong associations with non-Hodgkin's lymphomas (HR 2.86, 95% CI: 1.49, 5.49 for BMI⩾30 kg m^−2^) and cancers of the uterine corpus (HR 3.93, 95% CI: 2.35, 6.56 for BMI⩾35 kg m^−2^). Incidence of breast cancer was positively associated with high BMI only after age 65 years. These findings provide further evidence that overweight is associated with the incidence of several types of cancer.

Overweight and obesity is an increasing health problem, not only for industrialised countries but also for most other parts of the world. This epidemic appears to be affecting all ages, including childhood (WHO, 2000). Prospective studies have observed an association between overweight and overall mortality (Lew and Garfinkel, 1979; Manson *et al*, 1995; Calle *et al*, 1999), and several adverse health consequences of elevated body weight are well established, including type II diabetes, hypertension and coronary heart disease (Must *et al*, 1999). Obesity has also been associated with cancer incidence and mortality, and positive associations between obesity and risks of specific cancers, including endometrial and kidney cancer, are widely accepted (Calle and Kaaks, 2004). Inconsistent evidence of associations between other cancers and body weight may be due in part to small sample sizes and misclassification of body weight in retrospective studies. The relation between body mass index (BMI) and incidence of different cancers as ascertained by population-based cancer registries has been investigated by few studies (Moller *et al*, 1994; Wolk *et al*, 2001). We conducted a prospective investigation of the association between overweight and the incidence of cancer (overall and specific types) using data from the Vorarlberg cancer registry and a population-based cohort of more than 145 000 Austrian men and women followed for an average of nearly 10 years.

## MATERIALS AND METHODS

### Study population

The Vorarlberg Health Monitoring and Promotion Program (VHM&PP) is carried out in Vorarlberg, the westernmost province of Austria. It is performed routinely by the Agency of Social and Preventive Medicine and covers all adults of the whole province. The screening examination takes place in the practice of local physicians; it includes a physical examination, a blood test and a consultation with a doctor. Enrolment is voluntary and costs are covered by the participant's (compulsory) health insurance. More than two-thirds of the adult population of the province (between the age of 35 and 54 years) participated and underwent at least one examination since the beginning of the programme in 1985 (Ulmer *et al*, 2004). The VHM&PP has been described in detail previously (Ulmer *et al*, 2003).

Between 1985 and 2001, 167 371 adult Vorarlberg residents were enrolled in the VHM&PP Study Cohort after signing an informed consent to store and process personal data (height, weight, smoking and other factors). The current analysis was restricted to participants with complete data on height, weight and occupational group at enrolment. As in a previous study (Calle *et al*, 2003), participants with a baseline BMI (kg m^−2^) below normal (BMI<18.5 kg m^−2^, *n*=5053) were excluded. In order to avoid an influence of cancer growth on body weight, participants were further excluded if they had been diagnosed with a malignant cancer prior to enrolment, or within 1 year following enrolment (*n*=1831). Therefore, the first year of follow-up time was not considered in the analysis and participants with a follow-up period of less than 1 year (*n*=5528) were excluded. To evaluate the sensitivity of our analyses to this 1-year exclusion, we also repeated the analyses with a 3-year exclusion. The final study cohort consisted of 67 447 men and 78 484 women, with mean age at study entry of 41.8 years for men and 42.5 years for women (Table 1). The average time of follow-up was nearly 10 years, with a total of 1.45 million person-years. By the end of the total observation period of 17 years, 6241 incident cancers (other than nonmelanoma skin cancer) had been diagnosed (Table 1).

### Body mass index

Baseline height and weight were recorded by medical staff at enrolment during the VHM&PP physical examination. BMI was classified according to World Health Organisation guidelines as normal (18.50–24.99 kg m^−2^), overweight (25.00–29.99 kg m^−2^), obese class I (30.00–34.99 kg m^−2^), and obese class II and III (⩾35.00 kg m^−2^) (2000). Normal BMI was the reference category for all analyses, and obesity categories were combined when necessary to ensure a minimum of five cancer outcomes in each exposure group.

### Covariates

Associations were adjusted for smoking by including variables for current smoking and former smoking in the model, with the reference group being never smokers. Persons with missing smoking values were classified as never smokers because baseline questionnaire data did not differentiate between never-smokers and participants with missing values. However, smoking information from follow-up visits was available to validate the baseline smoking status of more than 70% of study participants. Occupational group (blue collar, white collar or self-employed) was determined by the insurance number of participants and was included in the models as a surrogate measure of socioeconomic status. Participants who were retired at baseline were classified according to their former occupation, and housewives according to their husband's occupation.

### End points

Between 1985 and 2002, incident invasive cancers were identified by the Vorarlberg cancer registry, which is accepted for publication by the International Agency for Research on Cancer (IARC) since 1993 (Parkin *et al*, 2003). The proportion of cancers discovered by death certificate only (DCO) in the Vorarlberg registry for cases diagnosed between 1993 and 1997 was 7% for men and 9% for women (Oberaigner *et al*, 2003), and for cases diagnosed between 1998 and 2002 about 5% in both sexes (W Oberaigner, personal communication, Cancer Registry of Tyrol). Nearly all cancers were histologically verified and coded according to the ninth revision of the *International Classification of Diseases* (ICD-9). Cohort data were linked with the Vorarlberg Death Index to identify deaths among cohort members to calculate person-years at risk.

### Statistical analysis

We used Cox proportional-hazards models to compute hazard rate ratios (HR) and 95% confidence intervals (95% CI) for overweight and obesity relative to normal BMI, adjusted for smoking and occupational group at baseline. The models included age (in single years) in the strata statement. In tests of linear trend by BMI, the median value for BMI within each interval was entered in a regression model, and the significance of the term tested by the Wald's *χ*^2^ test. All calculations were carried out with SAS version 8.2 software.

All analyses were performed separately for men and women. Analyses on specific cancers were restricted to those types of cancer with at least 50 cases in men or women.

## RESULTS

The study cohort consisted of 67 447 men and 78 484 women (Table 1). In men, positive linear trends in cancer incidence with increasing BMI were observed for colon and pancreatic cancer (Table 2). In comparison to men with normal weight, the hazard ratios for colon cancer were 1.56 (95% CI: 1.06, 2.30) for men with BMI 30.0–34.99 kg m^−2^, and 2.48 (1.15, 5.39) for BMI 35.0 kg m^−2^ or more. The hazard ratios for men with BMI of at least 30.0 kg m^−2^ were for rectal cancer 1.66 (1.00, 2.73) and for pancreatic cancer 2.34 (1.17, 4.66). Nonsignificant positive associations were found for kidney and liver cancers.

In women, there was a weak positive association between BMI and all cancers (Table 3). Endometrial (uterine corpus) cancer was strongly associated with obesity class I (hazard ratio 2.13 (1.38, 3.27)) and obesity class II and III (hazard ratio 3.93 (2.35, 6.56)) in comparison to normal weight. Furthermore, a positive association was found between obesity and the incidence of non-Hodgkin's lymphomas (NHL) (hazard ratio 2.86 (1.49, 5.49), with a BMI of at least 30.0 kg m^−2^). Kidney cancer was associated with overweight, though not statistically significant. In contrast to men, BMI was not associated with colon or rectal cancer in women.

There was little evidence of an association between breast cancer and BMI overall. However, breast cancer diagnosed in women aged 65 years or older was positively associated with BMI (hazard ratios 1.48 (1.12, 1.95) for obesity class I and 1.29 (0.79, 2.11) for obesity class II and III; *P* for trend 0.02).

All associations that were statistically significant after exclusion of the first year following entry into the study remained unchanged in terms of statistical significance when reanalysed excluding the first 3 years.

## DISCUSSION

The major strengths of our study are the prospective design, the large number of subjects, the coverage of incident cases and the length of follow-up. The population of Vorarlberg is culturally and ethnically rather homogenous, with more than 90% of Austrian origin (Ulmer *et al*, 2003). Body mass index was based on height and weight measured at initial physical examination. Incident cancers were ascertained by the population-based cancer registry and nearly all histologically confirmed; the likelihood of exposure and outcome misclassification was therefore low.

The limitations of our study include that, despite the overall size of the cohort, some cancers of interest (for example, oesophageal adenocarcinoma or gallbladder cancer) could not be evaluated due to small numbers of cases. In addition, the prevalence of obesity in our cohort was relatively low. Consequently the power to examine extreme levels of obesity, particularly in association with less common cancers, was limited. The high proportion of never-smoking patients with lung cancer (31%) suggests that there was some misclassification of smoking status, although this would probably attenuate the relation between BMI and most cancers, given the inverse association between smoking and body weight. We used occupational group as a rough surrogate for socioeconomic status, but were unable to account for such potentially confounding factors as alcohol consumption or physical activity.

Our finding of an association between BMI and both colon and rectum cancers in men supports earlier observations (Lew and Garfinkel, 1979; Giovannucci *et al*, 1995; Pan *et al*, 2004; Samanic *et al*, 2004) and may be due to the growth-promoting effects of insulin and insulin-like growth factor (IGF-1), both increased in obesity (Calle and Kaaks, 2004). Our failure to find an association between BMI and colon cancer in women also agrees with other studies (Phillips and Snowdon, 1985; Shimizu *et al*, 2003), and may be related to the protective effects of elevated oestrogen levels in overweight postmenopausal women, as found by studies of exogenous hormone therapy in such women (Calle *et al*, 1995; Newcomb and Storer, 1995).

We observed a positive association between pancreatic cancer and overweight or obesity in men, and to a lesser extent in women. Other studies have been inconsistent in this connection (Berrington *et al*, 2003). As with colon cancer, relations between pancreatic cancer and BMI have been attributed to the growth-promoting effects of elevated insulin and IGFs secondary to obesity (Takeda and Escribano, 1991), although the carcinogenic effects of insulin have also been proposed to explain positive associations with abnormal glucose metabolism (Gapstur *et al*, 2000) or diabetes mellitus (Everhart and Wright, 1995).

In line with previous reports (Yuan *et al*, 1998; Chow *et al*, 2000), we observed a positive relation between BMI and incidence of kidney cancer in men, which, however, did not reach statistical significance. In women, our findings showed a positive association between kidney cancer and overweight, but not obesity. Our finding of a positive association between BMI and breast cancer only among women at the age of 65 years or older is consistent with previous reports (Hunter and Willett, 1993).

A relation between endometrial cancer (cancer of the uterine corpus) and overweight is widely accepted (Calle and Kaaks, 2004), and it was found that this in very obese women (BMI⩾35 kg m^−2^) were more likely to be diagnosed with endometrial cancer than women with normal BMI at baseline (HR 3.93, 95% CI: 2.35, 6.56). A crucial pathway seems to be oestrogens that are not counterbalanced by progesterone (the ‘unopposed oestrogen’ hypothesis) (Kaaks *et al*, 2002). Anovulatory cycles in obese premenopausal women may contribute to a deficiency of progesterone, which normally opposes the mitogenic effect of oestrogen on the endometrial mucosa.

We also observed a strong positive association between BMI and NHL among women, but not men. Previous prospective studies of overweight and NHL have been inconsistent (Moller *et al*, 1994; Wolk *et al*, 2001; Cerhan *et al*, 2002; Calle *et al*, 2003; Samanic *et al*, 2004), though several case–control studies have reported an association between overweight and NHL in both sexes (Holly *et al*, 1999; Pan *et al*, 2004; Skibola *et al*, 2004). The incidence of NHL has increased in many parts of the world (Muller *et al*, 2005) and obesity might be a contributing factor.

As previously reported (Calle and Kaaks, 2004), liver cancer showed a (non-significant) association with BMI in men with point estimates clearly above one, but based on only 57 cases; there were too few cases of liver cancer for a separate analysis in women.

Many studies have examined relations between overweight and single cancer outcomes. Few prospective studies examined the influence of overweight on a range of specific cancers both in men and women (Lew and Garfinkel, 1979; Moller *et al*, 1994; Wolk *et al*, 2001; Calle *et al*, 2003). Our study of a large Austrian cohort provides additional support from another population for associations between BMI and the incidence of colon, rectal and pancreatic cancer, and to a lesser extent of kidney and liver cancer in men, and with endometrial cancer, postmenopausal breast cancer and NHL in women.

## Figures and Tables

**Table 1:** Characteristics of the VHM&PP study cohort

	**Men**	**Women**	**All**
Eligible VHM&PP participants (*N*)^a^	67 447	78 484	145 931
			
*Age at entry (years)*			
Mean (s.d.^b^)	41.78 (14.47)	42.48 (15.66)	42.16 (15.12)
Range	18.66–93.03	19.00–94.13	18.66–94.13
			
*Years of follow-up*			
Mean (s.d.^b^)	9.63 (4.63)	10.18 (4.56)	9.93 (4.60)
Range	0.00–16.89	0.00–16.84	0.00–16.89
Total person-years at risk	649 358	799 122	1 448 480
			
*BMI*^c^ (%)			
Normal: 18.5–24.9 kg m^−2^	50.47	62.86	57.13
Overweight: 25–29.9 kg m^−2^	40.05	25.60	32.28
Obese I: 30–34.9 kg m^−2^	8.23	8.55	8.40
Obese II and III: ⩾35 kg m^−2^	1.25	2.99	2.19
			
*Smoking* (%)			
Current smoker	29.99	20.78	25.03
Former smoker	13.27	4.99	8.79
			
*Occupational group*^d^ (%)			
Blue collar	37.29	38.09	37.72
White collar	51.99	54.08	53.12
Self-employed	10.72	7.82	9.16
			
Number of cancers	3337	2904	6241
			
*Age at cancer diagnosis (years)*			
Mean (s.d.^b^)	65.34 (11.49)	62.88 (13.21)	64.20 (12.38)
Range	22.87–95.69	22.43–96.29	22.43–96.29

VHM&PP=Vorarlberg Health Monitoring and Promotion Program; BMI=body mass index.

a Eligible participants were enrolled between 1985 and 2001, had complete baseline data for BMI, smoking and occupational group, and had no history of malignant cancer prior to or within 1 year after baseline. Participants with nonmelanoma skin cancer were excluded.

b s.d.: standard deviation.

c BMI: body mass index (kg m^−2^) based on height and weight measured at baseline physical examination.

d Occupational group classified according to insurance number for occupation at baseline, prior occupation (for pensioners) or husband's occupation (for housewives).

**Table 2:** Estimated HR and 95% CI for incident cancers diagnosed among male participants in the VHM&PP Study Cohort 1985–2001, according to BMI at enrolment

	**BMI (kg m^−2^)^a^**				
	**18.5–24.9**	**25–29.9**	**30–34.9**	**⩾35**	
**Type of cancer**	**Normal**	**Overweight**	**Obese I**	**Obese II and III**	
**Number of persons/py^b^**	**34 040/330 040**	**27 012/262 144**	**5552/50 385**	**843/6788**	***P* for trend**
*All cancers*					
Incident cases/py^b^	1364/10 293	1591/12 078	342/2459	40/259	
HR (95% CI)^c^	1.00	0.97 (0.91–1.05)	0.96 (0.85–1.08)	0.94 (0.69–1.29)	0.37
					
*Stomach cancer (ICD-9 151)*					
Incident cases/py^b^	58/400	75/546	13/67		
HR (95% CI)^c^	1.00	1.04 (0.73–1.47)	0.72^a^ (0.40–1.33)		0.44
					
*Colon cancer (ICD-9 153)*					
Incident cases/py^b^	86/663	128/942	39/275	7/44	
HR (95% CI)^c^	1.00	1.14 (0.86–1.50)	1.56 (1.06–2.30)	2.48 (1.15–5.39)	0.005
					
*Rectal cancer (ICD-9 154)*					
Incident cases/py^b^	45/327	69/499	24/163		
HR (95% CI)^c^	1.00	1.20 (0.82–1.75)	1.66^a^ (1.01–2.73)		0.053
					
*Liver cancer (ICD-9 155)*					
Incident cases/py^b^	18/128	29/197	10/92		
HR (95% CI)^c^	1.00	1.32 (0.73–2.37)	1.67^a^ (0.75–3.72)		0.19
					
*Pancreatic cancer (ICD-9 157)*					
Incident cases/p^b^	19/129	31/250	14/109		
HR (95% CI)^c^	1.00	1.29 (0.73–2.27)	2.34^a^ (1.17–4.66)		0.02
					
*Lung cancer (ICD-9 162)*					
Incident cases/py^b^	209/1427	198/1288	50/308	7/32	
HR (95% CI)^c^	1.00	0.80 (0.66–0.97)	0.88 (0.65–1.20)	0.88 (0.41–1.86)	0.15
					
*Melanoma (ICD-9 172)*					
Incident cases/py^b^	59/373	56/409	7/48		
HR (95% CI)^c^	1.00	1.00 (0.68–1.46)	0.59^a^ (0.27–1.31)		0.32
					
*Prostate cancer (ICD-9 185)*					
Incident cases	446/4001	583/5165	99/766	10/88	
HR (95% CI)^c^	1.00	1.03 (0.91–1.17)	0.82 (0.66–1.03)	0.73 (0.39–1.37)	0.16
					
*Bladder cancer (ICD-9 188)*					
Incident cases/py^b^	78/522	78/507	19/136		
HR (95% CI)^c^	1.00	0.81 (0.59–1.11)	0.74^a^ (0.45–1.22)		0.15
					
*Kidney cancer (ICD-9 189)*					
Incident cases/py^b^	46/356	70/486	21/162		
HR (95% CI)^c^	1.00	1.19 (0.82–1.74)	1.46^a^ (0.87–2.46)		0.14
					
*Non-Hodgkin's lymphoma (ICD-9 200*+*202)*					
Incident cases/py^b^	31/236	45/288	8/54		
HR (95% CI)^c^	1.00	1.26 (0.80–2.01)	0.91^a^ (0.41–1.99)		0.86

VHM&PP=Vorarlberg Health Monitoring and Promotion Program; HR=hazards ratio; CI=confidence interval; ICD=International Classification of Diseases; BMI=body mass index.

a Obese categories (class I and class II and III) were combined as needed to ensure at least five cases in each.

b Person-years.

c The Cox proportional-hazards model was stratified according to age at enrolment (in years) and adjusted for smoking status and occupational group.

**Table 3:** Estimated HR and 95% CI for incident cancers diagnosed among female participants in the VHM&PP Study Cohort 1985–2001, according to BMI at enrolment

	**BMI (kg m^−2^)^a^**				
**Type of cancer**	**18.5–24.9**	**25–29.9**	**30–34.9**	**⩾35**	
**Number of persons/py^b^**	**49 336/502 849**	**20 090/208 574**	**6709/66 351**	**2349/21 349**	***P* for trend**
*All cancers*					
Incident cases/py^b^	1425/10 712	997/6883	369/2493	113/795	
HR (95% CI)^c^	1.00	1.05 (0.96–1.14)	1.16 (1.03–1.30)	1.18 (0.97–1.43)	0.008
					
*Stomach cancer (ICD-9 151)*					
Incident cases/py^b^	56/394	36/212	20/146	6/45	
HR (95% CI)^c^	1.00	0.78 (0.51–1.20)	1.28 (0.76–2.15)	1.34 (0.57–3.13)	0.48
					
*Colon cancer (ICD-9 153)*					
Incident cases/py^b^	122/958	106/773	35/238	8/82	
HR (95% CI)^c^	1.00	1.13 (0.86–1.47)	1.11 (0.76–1.62)	0.88 (0.43–1.81)	0.73
					
*Rectal cancer (ICD-9 154)*					
Incident cases/py^b^	68/504	48/315	12/100	5/27	
HR (95% CI)^c^	1.00	0.90 (0.62–1.31)	0.66 (0.36–1.23)	0.96 (0.38–2.39)	0.32
					
*Pancreatic cancer (ICD-9 157)*					
Incident cases/py^b^	29/231	21/154	15/80		
HR (95% CI)^c^	1.00	0.87 (0.49–1.53)	1.42^a^ (0.76–2.68)		0.4
					
*Lung cancer (ICD-9 162)*					
Incident cases/py^b^	64/513	45/300	17/97		
HR (95% CI)^c^	1.00	1.00 (0.68–1.48)	0.87^a^ (0.50–1.50)		0.67
					
*Melanoma (ICD-9 172)*					
Incident cases/py^b^	79/535	38/268	13/77		
HR (95% CI)^c^	1.00	1.03 (0.68–1.54)	0.86^a^ (0.47–1.57)		0.72
					
*Breast cancer (ICD-9 174)*					
Incident cases/py^b^	551/4162	335/2326	123/860	36/270	
HR (95% CI)^c^	1.00	0.96 (0.83–1.10)	1.07 (0.88–1.31)	1.01 (0.72–1.42)	0.8
					
*Cervical cancer (ICD-9 180)*					
Incident cases/py^b^	41/205	17/106	6/43		
HR (95% CI)^c^	1.00	0.85 (0.47–1.54)	0.69^a^ (0.29–1.66)		0.37
					
*Cancer of the uterine corpus (ICD-9 182)*					
Incident cases/py^b^	63/452	59/441	33/230	20/93	
HR (95% CI)^c^	1.00	1.29 (0.90–1.86)	2.13 (1.38–3.27)	3.93 (2.35–6.56)	<0.001
					
*Ovarian cancer (ICD-9 183)*					
Incident cases/py^b^	61/490	39/245	21/141		
HR (95% CI)^c^	1.00	1.03 (0.68–1.56)	1.25^a^ (0.75–2.08)		0.44
					
*Bladder cancer (ICD-9 188)*					
Incident cases/py^b^	21/128	22/120	11/85		
HR (95% CI)^c^	1.00	1.35 (0.74–2.48)	1.60^a^ (0.76–3.36)		0.19
					
*Kidney cancer (ICD-9 189)*					
Incident cases/py^b^	32/290	44/299	12/77		
HR (95% CI)^c^	1.00	1.81 (1.13–2.89)	1.14^a^ (0.58–2.24)		0.3
					
*Thyroid cancer (ICD-9 193)*					
Incident cases/py^b^	29/173	24/183	8/52		
HR (95% CI)^c^	1.00	1.45 (0.82–2.58)	1.18^a^ (0.53–2.65)		0.44
					
*Non-Hodgkin's lymphoma (ICD-9 200*+202)					
Incident cases/py^b^	22/154	24/170	18/126		
HR (95% CI)^c^	1.00	1.64 (0.89–3.01)	2.86^a^ (1.49–5.49)		0.002

VHM&PP=Vorarlberg Health Monitoring and Promotion Program; HR=hazards ratio; CI=confidence interval; ICD=International Classification of Diseases; BMI=body mass index.

a Obese categories (class I and class II and III) were combined as needed to ensure at least five cases in each.

b Person-years.

c The Cox proportional-hazards model was stratified according to age at enrolment (in years) and adjusted for smoking status and occupational group.
